# The impacts of dietary sphingomyelin supplementation on metabolic parameters of healthy adults: a systematic review and meta-analysis of randomized controlled trials

**DOI:** 10.3389/fnut.2024.1363077

**Published:** 2024-02-23

**Authors:** Chen-Zi Li, Li-Mei Wu, Chen-Xi Zhu, Huan-Yu Du, Guo-Xun Chen, Fang Yang

**Affiliations:** ^1^School of Laboratory Medicine, Hubei University of Chinese Medicine, Wuhan, China; ^2^College of Food Science and Technology, Huazhong Agricultural University, Wuhan, China

**Keywords:** sphingomyelin, metabolic parameters, randomized controlled trials, meta-analysis, protective effect

## Abstract

**Background:**

Studies have shown that sphingomyelin (SM) and its metabolites play signaling roles in the regulation of human health. Endogenous SM is involved in metabolic syndrome (MetS), while dietary SM supplementation may maintain lipid metabolism and prevent or alleviate MetS. Therefore, we hypothesized that dietary SM supplementation is beneficial for human health.

**Aims:**

In order to examine the impacts of dietary SM on metabolic indexes in adults without MetS, we performed a meta-analysis to test our hypothesis.

**Methods:**

A comprehensive search was performed to retrieve randomized controlled trials that were conducted between 2003 and 2023 to examine the effects of dietary SM supplementation on metabolic parameters in the Cochrane Library, PubMed, Web of Science, Embase, and ClinicalTrials.gov databases. RevMan 5.4 and Stata 14.0 software were used for meta-analysis, a sensitivity analysis, the risk of bias, and the overall quality of the resulted evidence.

**Results:**

Eventually, 10 articles were included in this meta-analysis. Dietary SM supplementation did not affect the endline blood SM level. When compared to the control, SM supplementation reduced the blood total cholesterol level [MD: −12.97, 95% CI: (−14.57, −11.38), *p* < 0.00001], low-density lipoprotein cholesterol level [MD: −6.62, 95% CI: (−10.74, −2.49), *p* = 0.002], and diastolic blood pressure [MD: −3.31; 95% CI (−4.03, −2.58), *p* < 0.00001] in adults without MetS. The supplementation also increased high-density lipoprotein level [MD:1.41, 95% CI: (0.94, 1.88), *p* < 0.00001] and muscle fiber conduction velocity [MD: 95% 1.21 CI (0.53, 1.88), *p* = 0.0005]. The intake of SM had no effect on the blood phospholipids and lyso-phosphatidylcholine, but slightly decreased phosphatidylcholine, phosphatidylethanolamine, and phosphatidylinositol concentrations. Dietary SM supplementation reduced insulin level [MD: −0.63; 95% CI (−0.96, −0.31), *p* = 0.0001] and HOMA-IR [MD: −0.23; 95% CI (−0.31, −0.16), *p* < 0.00001] without affecting blood levels of glucose and inflammatory cytokines.

**Conclusion:**

Overall, dietary SM supplementation had a protective effect on blood lipid profiles and insulin level, but had limited impacts on other metabolic parameters in adults without MetS. More clinical trials and basic research are required.

**Systematic review registration:**

PROSPERO, identifier CRD42023438460.

## 1 Introduction

Metabolic syndrome (MetS), also known as syndrome X, is a pathological condition characterized by abdominal obesity, insulin resistance, hypertension, and hyperlipidemia (1). Approximately 20 to 25% of adults in the world are affected by MetS (2). In the meantime, the prevalence of MetS resulting from obesity in children and adolescents is also on the rise (3, 4). MetS has the potential to induce metabolic disorders in the body, which is a risk factor not only for cardiovascular diseases (CVDs), type 2 diabetes mellitus (T2DM), non-alcoholic fatty liver disease (NAFLD) and other chronic metabolic diseases, but also for cancer and all-cause mortality (5–7). As CVDs are the primary cause of mortality on a global scale, it has become imperative to investigate the impact of MetS in order to alleviate the substantial burden of CVDs (8). In conjunction with genetic and epigenetic influences, some lifestyle and environmental variables, such as excessive caloric intake and sedentary behavior, have been recognized as significant determinants in the pathogenesis of MetS (8, 9). Identifying clinical high-risk factors for MetS and finding early intervention methods to prevent the occurrence and progression of severe complications have significant public health implications. According to World Health Organization, European group for study of insulin resistance, International Diabetes Federation, American Heart Association, and National Cholesterol Education Program Adult Treatment Panel III, the main diagnostic criteria of MetS are central obesity (elevated waist circumference), elevated blood glucose level, elevated triglyceride (TG) and total cholesterol (TC) levels, reduced high density lipoprotein cholesterol (HDL-C) level, and elevated blood pressure (8). The prevention and treatment of MetS often involve individualized therapies targeting dyslipidemia, hyperglycemia, and hypertension, with food restriction and regular exercise (10).

Blood lipid profiles such as levels of TG, cholesterol, phospholipids (PLs) and fatty acids, particularly those related to cholesterol homeostasis, are the essential indexes for controlling MetS (11). The plasma low density lipoprotein cholesterol (LDL-C) is maintained though the intestinal cholesterol absorption, endogenous cholesterol synthesis, and cholesterol clearance (12). Sphingomyelin (SM) is a sphingolipid found in animal tissues, which consists of a phosphorylcholine head group, a long-chain fatty acyl group and a sphingosine (13). It predominantly colocalizes with cholesterol on the outer leaflet of the plasma membrane, lysosomal and Golgi membranes, as well as in lipoproteins (14, 15). SM and its metabolites, including sphingoid bases, ceramide (Cer), ceramide-1-phosphate (C1P), and sphingosine-1-phosphate (S1P), play an important role in human health (10). Several investigations have shown that the endogenous SM and its metabolites are involved in the pathological processes associated with obesity, diabetes, and atherosclerosis (16–18). The amount of endogenous SM in the plasma is associated with atherosclerosis, which is considered a risk factor for CVDs (19). It has been shown that atherosclerotic LDL contains 10–50 times more Cer than the control ones (20). Patients with coronary heart disease showed higher plasma SM levels than the control subjects in case–control studies and multi-ethnic cohort studies (14, 21, 22). SM and Cer species containing palmitate exhibited the strongest positive correlation with cardiovascular and total mortality (23). Circulating C16-SM and C16-Cer, the potential biomarkers, have been associated with CVDs in people with T2DM (24, 25). However, one study shows that in adults with T2DM, SM containing a very long chain saturated fatty acid is associated with a reduced risk of CVDs (25). Nevertheless, Yeboah et al. discovered that plasma SM levels did not serve as a predictor of incidence of coronary heart disease events after a 5-year follow-up study (26).

However, the potential impact of dietary SM on the regulation of cholesterol homeostasis, lipid metabolism, and the mitigation of symptoms associated with obesity, diabetes, and atherosclerosis has not been revealed and is of significant interest. Studies on animals have shown that the dietary SM supplementation prevents atherosclerosis through the inhibition of cholesterol absorption (27–30), modification of plasma and hepatic cholesterol and TG metabolism (31, 32), formation of lipoproteins and intestinal mucosal development (33). Dietary SM derived from milk and egg yolk diminished the high-fat diet (HFD)-induced hepatic steatosis by controlling lipid absorption and metabolism, and reduced blood lipopolysaccharide via bifidogenic effects and changes in the distal gut microbiota (34, 35). Then, whether dietary SM has a positive impact on human health is still an open question. The average Western diet provides humans with 300 mg to 400 mg of sphingolipids daily, and the majority of them is SM found in meat, milk, egg products and fish (36). The extent to which dietary SM influences the endogenous sphingolipidome is still unknown. Ohlsson et al. first designed a parallel study to examine the effects of the consumption of buttermilk fortified with SM on plasma lipids over 4 weeks, and did not observe the lipid-lowering benefits of SM-enriched milk polar lipids. However, the results suggest that 700 mg/day of dietary SM mitigated the rise in TG and LDL levels associated with an increase in calorie and fat consumption (37). According to Ramprasath et al., with the exception of an increase in HDL, the addition of 1 g/day of SM to the diet has no effect on cholesterol absorption, synthesis, and the blood lipid profile in a crossover randomized controlled trial (RCT) (38). No clinically significant changes in body mass index (BMI), glucose, blood lipid profile and liver function of healthy adults after the dietary SM supplementation were observed in subsequent RCTs (39–42). Therefore, we assume that dietary SM intake is beneficial to human body. In order to test this hypothesis, we conducted a meta-analysis using data retrieved from 10 RCT studies to obtain a more definite conclusion about the effect of dietary SM supplementation on relevant biomarkers of MetS.

## 2 Methods

### 2.1 Scheme and registration

The present systematic review was filed in PROSPERO (CRD42023438460) and carried out in accordance with the Preferred Reporting Items for Systematic Reviews and Meta-Analyses (PRISMA) statement (43). For the current study, ethical approval was not required.

### 2.2 Search strategy

The search strategy took into account three primary concepts, SM, PLs, and adults. For each concept, Medical Subject Headings and keywords were mapped. Subsequently, a search was conducted in the PubMed database to get a broader range of search terms, which included SM, PLs, gangliosides, sphingolipids, Cer, sphingosine, milk, egg, along with adults. RCTs on the effects of dietary SM on adults were searched in PubMed, Web of Science, Embase, ClinicalTrials.gov, and Cochrane Library databases from January 2003 to October 2023, with a language restriction to English. The search process and the related results were shown in Supplementary material 1. To ensure the comprehensiveness of reference lists, the cited references of the included relevant studies were also carefully reviewed. Four team members were divided into two groups: LMW and HYD, and CZL and CXZ. These two groups independently conducted reference screening in pairs and later compared the titles and/or abstracts of the publications that they retrieved. Subsequently, a comprehensive assessment was conducted on the whole texts of possibly eligible research in order to identify papers that satisfied the predetermined criteria for inclusion and exclusion. To ensure the integrity of the research selection process, a calibration exercise was first conducted. A consensus was achieved either via the resolution of conflicts or with the aid of another reviewer (FY).

### 2.3 Inclusion and exclusion criteria

The following inclusion criteria were used to ensure that the studies were appropriately selected. (1) The effects of SM supplementation on metabolic parameters of adults without MetS were studied. (2) RCTs studies should have compared SM supplementation with a placebo. (3) At least one MetS component (anthropometric parameters, blood pressure measurement, blood lipid and glycemic profile) was investigated in RCTs. (4) Only RCTs lasting at least 2 weeks were included to ensure that the interventions had enough time to have an effect. (5) The data presented in the selected studies should be continuous measures, as the mean ± standard deviation (SD) of the baseline and final value, and 95% confidence interval (CI) of the indicators must also be provided. (6) The language was limited to English. Articles that did not provide information on the amount of SM consumed and specific physical indicators were excluded. Studies were excluded if their subjects are minors, elderly people, unhealthy people with MetS, atherosclerosis, CVDs, diabetes and other chronic metabolic diseases, or acute diseases such as appendicitis, myocardial infarction, and cerebral hemorrhage. Furthermore, studies with an intervention duration of less than 2 weeks or longer than 13 weeks should be precluded, as the duration of the intervention is decisive. Finally, studies that were published as letters, meeting abstracts, meta-analyses, or reviews were excluded. If there were duplicate studies, we included only the most recent or complete one.

### 2.4 Data extraction

The following extracted data were collected from all eligible studies, including fundamental characteristics (the first author’s name, year of publication, country, type of RCT, representation of population and sources of funding), diagnosis criteria of MetS, sample size, the age, gender, and conditions of the subjects, baseline data of participants, control and intervention (the dosage of SM and duration time), outcome measures, and records used for assessing bias risk. The outcome indicators included: (1) blood SM, (2) anthropometric parameters including BMI, body fat percentage (BF%), knee extension, muscle fiber conduction velocity (MFCV), systolic blood pressure (SBP) and diastolic blood pressure (DBP), (3) blood lipid profile including TC, TG, LDL-C, HDL-C and LDL-C/HDL-C, apolipoprotein B (ApoB) and apolipoprotein A1 (ApoA1), (4) blood PL profile including PLs, phosphatidylcholine (PC), lyso-phosphatidyl choline (Lyso-PC), phosphatidylethanolamine (PE) and phosphatidylinositol (PI), (5) blood glycemic indexes including glucose, insulin and homeostasis model assessment of insulin resistance (HOMA-IR), (6) inflammatory response factors including aspartate transaminase (AST), alanine transaminase (ALT) and C-reactive protein (CRP). To determine if any data were missing or inadequate, we attempted to contact the authors of the listed papers. Prior to validating the data extraction process, a calibration procedure was performed. Data were cross-checked, and any discrepancies were resolved through discussion with the two authors (FY and GXC).

### 2.5 Quality assessment of studies

Two researchers (CZL, LMW) assessed the risk of bias in the included RCTs independently and consistently using the Cochrane criteria (44). Any controversy regarding literature deviation risk was resolved through discussion and consultation with the two authors (FY and GXC). The risk of bias in the included RCTs was assessed, including random sequence generation, allocation concealment, blindness of participants and personnel, blindness of outcome assessment, incomplete outcome data, selective reporting bias, and other biases. Grading of Recommendations Assessment, Development, and Evaluation (GRADE) methodology was used to assess the overall quality of the evidence generated by the meta-analysis, taking into account high risk of bias, imprecision, indirectness, heterogeneity, and publication bias.^1^ Supplementary material 2 shows that the overall certainty of evidence was rated as “very low,” “low,” “moderate” or “high.”

### 2.6 Data synthesis and statistical analysis

To extract the baseline and final values of the indicators mentioned above, unifying the index nomenclature and units in the involved articles was needed. Using the following conversion factors to unify the units of the indicators: 1 μg/100 ul = 0.01 mg/mL for SM, 1 mmo/L = 38.66 mg/dL for TC, HDL-C, and LDL-C, 1 mmol/L = 88.6 mg/dL for TG, 1 mmol/L = 18 mg/dL for glucose, 1 pmol/L = 0.167 mIU/L for insulin, and 1 mg/dL = 10 mg/L for CRP. For PLs, PC, PE, PI and Lyso-PC, 1 μg/100ul = 0.01 mg/mL. The statistical analyses to perform a meta-analysis and the funnel plots to evaluate publication bias were performed by RevMan software (version 5.4; Cochrane, London, United Kingdom) and Stata software (version 14.0; StataCorp, Texas, United States). The effects of SM supplementation were described by the mean difference (MD) with 95% CI. *p* < 0.05 was considered as statistically significant. Before stratifying the indicators by dose, statistical heterogeneity across the included studies was assessed by using the *I*^2^ value with 50% or higher regarded as high (45). In consideration of the heterogeneity of the included studies, effects models were chosen. The random-effects model was utilized to aggregate the data if *I*^2^ > 50% and *p* < 0.05. Otherwise, the fixed-effects model was applied. In order to elucidate the underlying factors contributing to the observed heterogeneity, a subgroup analysis was performed based on the dosage of SM supplementation. Prescribed by stringent criteria for inclusion and exclusion, and based on the average daily intake of 300 mg to 400 mg sphingolipids (36), the subjects were divided into high and low dose SM intervention groups. The high dose group had a dose greater than 400 mg/day, while the low dose group had a dose less than or equal to 400 mg/day. Sensitivity analysis was used to determine the robustness and stability of the meta-analysis results by excluding (1) studies with a high risk of bias and (2) numerical outliers. Evidence of publication bias was assessed with the Egger’s test using Stata if ten or more studies were included in each meta-analysis.

## 3 Results

### 3.1 Search results

**Figure 1:**
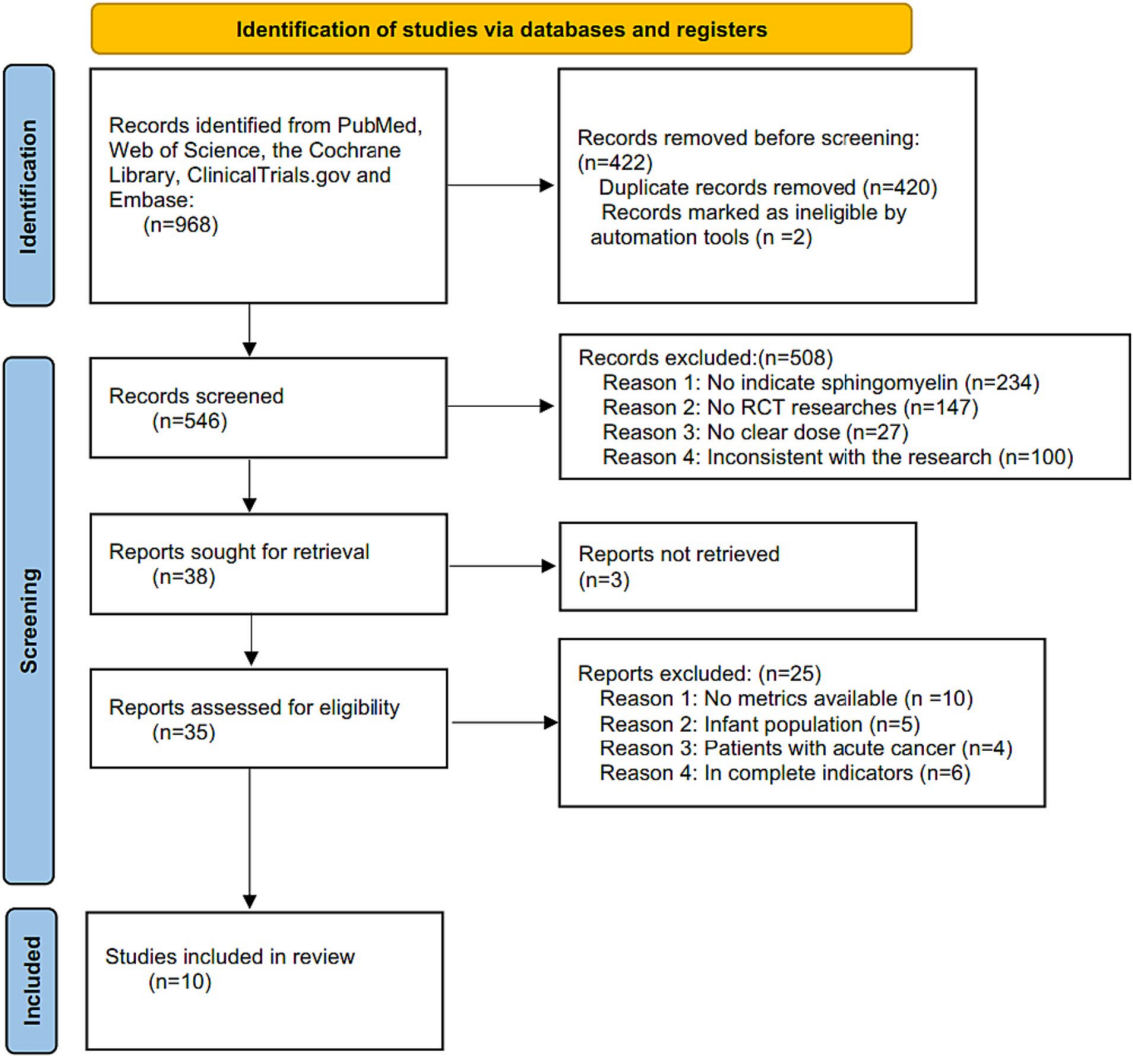
Flow chart of retrieval process included in meta-analysis.

The flowchart of retrieval process is shown in Figure 1, a total of 968 articles were retrieved from the 5 databases mentioned above, no more articles were found when searching the references’ list. In all, 546 articles were examined after the removal of duplicates and records marked as ineligible by automation tools. Based on the titles and abstracts, an additional 508 studies were excluded as they were non-SM (*n* = 234), non-clinical (*n* = 147), no clear dosage (*n* = 27) and inconsistent with the aim of this research (*n* = 100). In addition, there were 3 articles with incomplete retrieval reports. Eventually, 35 full text articles left, which 25 of them were excluded based on the lack of analyzable indicators (*n* = 10), infant participants (*n* = 5), patients with acute cancer (*n* = 4), and incomplete indicators (*n* = 6). Ultimately, a total of 10 RCT articles (37, 39–42, 46–50) were included in the final systematic review and meta-analysis with a total of 458 study subjects.

### 3.2 Quality assessment of the studies

**Figure 2:**
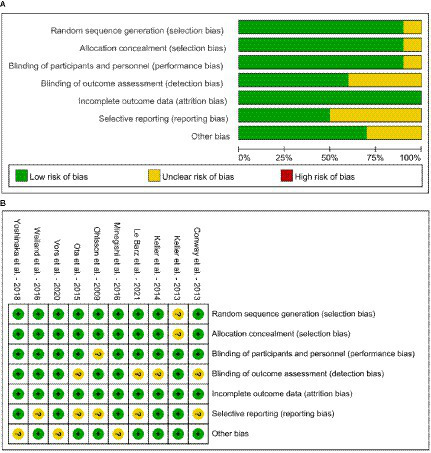
Risk of bias graph of included studies **(A)** risk of bias graph and **(B)** risk of bias summary.

According to Cochrane’s criteria, the risk assessment of the included studies is shown in Figure 2. Overall, the quality of the articles is good. Only 1 article did not specify whether randomization grouping was used (47). For group concealment, 9 articles used the group concealment method in the experimental process, while only 1 article did not use it (47). Similarly, only 1 article in the included studies did not use the double-blind method (37). We also assessed whether there was bias in the study results. The quality of the studies was evaluated in terms of completeness of results, reporting bias, and information bias. All the studies had complete outcome data. Only 4 studies did not clearly describe the blinding of outcome assessment (39, 40, 46, 48). Five studies found that there were no reporting bias from various aspects (39, 41, 47, 49, 50), while 3 studies identified potential biases (41, 49, 50). These three articles are considered to pose other bias, such as information bias and measure bias. The GRADE evidence quality of the included studies is shown in Supplementary material 2. Among the 35 outcome indicators in the total, low and high dose subgroups of the same indicator, 20 indicators (SM, BF%, knee extension, DBP, TC-high dose subgroup, LDL-C-high dose subgroup, HDL-C-high dose subgroup, LDL-C/HDL-C, LDL-C/HDL-C low dose subgroup, LDL-C/HDL-C high dose subgroup, ApoA, ApoB, PL, PC, Lyso-PC, insulin, HOMA-IR, AST, ALT and CRP) were of high quality and 14 indicators (BMI, MFCV, SBP, TC, TC-low dose subgroup, TG, TG low dose subgroup, TG high dose subgroup, LDL-C, LDL-C low dose subgroup, HDL-C, HDL-C low dose subgroup, PE, PI and glucose) were of medium quality. Taking the results of Cochrane’s criteria and GRADE evidence together, the overall quality of this meta-analysis was of high quality.

### 3.3 Characteristics results of the included RCTs

The basic characteristics of the included studies with RCT design are shown in Table 1. A total of 448 participants were included in this meta-analysis and were published between 2003 and 2023. Among the 10 studies included, one survey was conducted in Switzerland, four in Japan, one in Canada, two in Germany, and two in France. Four of these investigations were reported from Asian countries, the others reports were from North America and European countries. All studies were conducted in developed countries. The SM supplementation was orally administered, without other conversion forms. The experimental group was given different dosages of milk SM, while the control group was given placebo with the same durations time. Confounding factors including age and gender were controlled in 10 studies. The summary of outcomes and results obtained from the 10 included RCTs are detailed in Table 2.

**Table 1:** Information of included studies.

Included studies	Country	NO. (I/C)	Treatment group	Control group	Daily Dose Equivalent (mg)	Duration Time (d)	Clinical outcomes
Ohlsson et al. (37)	Sweden	29/19	SM	Placebo	700	28 days	③④⑮⑯⑰⑱⑲
Keller et al. (47)	Japan	14/0	SM	Placebo	700 or 1,400	30 days	⑦⑧⑨⑩⑪⑫⑬⑭⑮⑯⑰
Conway et al. (46)	Canada	17/17	SM	Placebo	23.62	56 days	⑬⑭⑮⑯⑰
Keller et al. (39)	German	19/20	SM	Placebo	750	42 days	①②③④⑬⑭ ⑮⑯⑰㉕
Ota et al. (40)	Japan	22/22	SM	Placebo	38.1	70 days	⑤⑥⑬⑭⑱⑳㉓㉔
Minegishi et al. (49)	Japan	11/11	SM	Placebo	80.3	70 days	④⑤⑥
Weiland et al. (42)	German	31/31	SM	Placebo	462	56 days	①②③④⑬⑭⑮⑯⑰⑱⑲⑳㉑㉒㉓㉔㉕
Yoshinaka et al. (50)	Japan	36/35	SM	Placebo	38.1	56 days	③④⑤
Vors et al. (41)	France	38/18	SM	Placebo	24 or 65	28 days	①②③⑬⑭⑮ ⑯⑰⑱⑲⑳㉑㉒
Barz et al. (48)	France	39/19	SM	Placebo	750 or 1,250	28 days	⑦⑧⑨⑩⑪⑫

No., numbers of participants; I, intervention group; C, control group; Outcome Indicators: ① SBP, systolic blood pressure; ② DBP, diastolic blood pressure; ③ BMI, body mass index; ④ BF%, body fat percentage; ⑤ Knee extension; ⑥ MFCV, muscle fiber conduction velocity; ⑦ SM, sphingomyelin; ⑧ PLs, total phospholipids; ⑨ Lyso-PC, Lyso-phosphatidyl cholines; ⑩ PC, phosphatidyl cholines; ⑪ PE, phosphatidyl-ethanolamine; ⑫ PI, phosphatidyl-inositol; ⑬ TC, total cholesterol; ⑭ TG, triglyceride; ⑮ LDL-C, low-density lipoprotein cholesterol; ⑯ HDL-C, high-density lipoprotein cholesterol; ⑰ LDL-C/HDL-C; ⑱ ApoA, apolipoprotein A; ⑲ ApoB, apolipoprotein B; ⑳ Glucose; ㉑ insulin; ㉒ HOMA-IR, homeostasis model assessment of insulin resistance; ㉓ AST, aspartate transaminase; ㉔ ALT, alanine transaminease; ㉕ CRP, C-reactive protein.

**Table 2:** Outcomes and results of included studies.

First Author, year	Baseline SM	Endline SM	Baseline Outcomes	Endline Outcomes	Conclusion
Ohlsson et al., 2009 (37)			TC: I: male: 187.89 ± 7.73 female: 181.70 ± 11.21 C: male: 173.58 ± 8.89 female: 172.81 ± 8.89	TC: I: male: 189.05 ± 5.41 female: 179.77 ± 5.41 C: male:177.06 ± 15.46 female: 178.59 ± 20.10	Increased daily SM consumption had no discernible effect on fasting plasma lipids or lipoprotein levels.
			TG: I: male: 101.89 ± 13.29 female: 81.51 ± 11.51 C: male: 85.06 ± 10.63 female: 59.36 ± 6.20	TG: I: male: 101.89 ± 7.08 female: 88.68 ± 27.47 C: male: 101.00 ± 35.44 female: 67.96 ± 13.29	
			LDL-C: I: male: 127.96 ± 6.96 female: 119.46 ± 10.44 C: male: 114.05 ± 8.51 female: 103.61 ± 5.80	LDL-C: I: male: 129.51 ± 11.21 female: 115.59 ± 3.87 C: male: 119.07 ± 10.82 female: 107.86 ± 15.07	
			HDL-C: I: male: 51.42 ± 2.71 female: 57.99 ± 3.48 C: male: 51.80 ± 3.09 female: 69.59 ± 7.73	HDL-C: I: male: 51.42 ± 3.48 female: 57.26 ± 4.41 C: male: 52.58 ± 5.41 female: 72.68 ± 10.05	
			LDL-C/HDL-C: I: male: 2.49 ± 2.57 female: 2.06 ± 3.00 C: male: 2.20 ± 2.75 female: 1.49 ± 0.75	LDL-C/HDL-C: I: male: 2.42 ± 3.22 female: 2.03 ± 0.88 C: male: 2.27 ± 1.93 female: 1.48 ± 1.50	
			ApoB: I: male: 0.88 ± 0.05 female: 0.82 ± 0.06 C: male: 0.79 ± 0.05 female: 0.69 ± 0.03	ApoB: I: male: 0.88 ± 0.03 female: 0.78 ± 0.01 C: male: 0.80 ± 0.08 female: 0.72 ± 0.01	
			ApoA 1: I: male:1.39 ± 0.03 female: 1.51 ± 0.07 C: male: 1.37 ± 0.05 femalee:1.60 ± 0.04	ApoA 1: I: male:1.34 ± 0.04 female: 1.48 ± 0.04 C: male: 1.38 ± 0.09 female: 1.58 ± 0.07	
Keller et al., 2013 (47)	C: 0.46 ± 0.08	700 mg/d: 0.45 ± 0.10 1,400 mg/d: 0.48 ± 0.07	TC: C: 197.93 ± 24.36	TC: I: 700 mg/d: 182.47 ± 27.44 I: 1400 mg/d: 196.01 ± 30.54	After SM consumption, there was no significant change in TC, TG, LDL-C concentration and the LDL/HDL ratio compared to baseline.
			TG: C: 93.92 ± 18.60	TG: I: 700 mg/d: 95.68 ± 26.58 I: 1400 mg/d: 98.35 ± 31.89	
			LDL-C: C: 104.76 ± 29.76	LDL-C: I: 700 mg/d: 98.19 ± 26.67 I: 1400 mg/d: 110.56 ± 32.47	
			HDL-C: C: 68.04 ± 18.17	HDL-C: I: 700 mg/d: 62.62 ± 15.46 I: 1400 mg/d: 64.94 ± 16.23	
			LDL-C/HDL-C: C: 1.74 ± 1.02	LDL-C/HDL-C: I: 700 mg/d: 1.72 ± 0.86 I: 1400 mg/d: 1.86 ± 0.93	
			PLs: C: 2.23 ± 0.34	PLs: I: 700 mg/d: 2.27 ± 0.44 I: 1400 mg/d: 2.35 ± 0.36	
			Lyso-PC: C: 0.07 ± 0.04	Lyso-PC: I: 700 mg/d: 0.08 ± 0.03 I: 1400 mg/d: 0.08 ± 0.05	
			PC: C: 1.58 ± 0.24	PC: I: 700 mg/d: 1.59 ± 0.36 I: 1400 mg/d: 1.65 ± 0.30	
			PE: C: 0.09 ± 0.04	PE: I: 700 mg/d: 0.12 ± 0.05 I: 1400 mg/d: 0.11 ± 0.05	
			PI: C: 0.03 ± 0.01	PI: I: 700 mg/d: 0.03 ± 0.01 I: 1400 mg/d: 0.03 ± 0.01	
Conway et al., 2013 (46)			TC: C: 227.32 ± 35.95	TC: I: 221.91 ± 31.31 C: 228.87 ± 36.34	The consumption of dietary SM may potentially lead to.decreased the serum TC, LDL-C and TG levels in both men and women, primarily by impeding the absorption of cholesterol into the intestines
			TG: C: 107.21 ± 52.27	TG: I: 102.78 ± 41.64 C: 115.18 ± 53.16	
			LDL-C: C: 144.98 ± 26.68	LDL-C: I: 138.79 ± 24.74 C: 143.43 ± 27.45	
			HDL-C: C: 60.70 ± 18.56	HDL-C: I: 62.63 ± 16.62 C: 62.63 ± 18.17	
			LDL-C/HDL-C: C: 2.38 ± 1.43	LDL-C/HDL-C: I: 2.21 ± 1.49 C: 2.29 ± 1.51	
Keller et al., 2014 (39)	I: 0.63 ± 0.13 C: 0.61 ± 0.11	I:0.58 ± 0.08 C:0.57 ± 0.10	TC: I: 172.04 ± 21.65 C: 170.10 ± 22.43	TC: I: 172.81 ± 24.74 C: 178.60 ± 23.19	There was no significant difference in plasma SM and lipid profile after the dietary SM consumption.
			TG: I: 93.91 ± 44.3 C: 94.80 ± 43.41	TG: I: 93.03 ± 37.21 C: 92.14 ± 37.21	
			LDL-C: I: 92.78 ± 19.71 C: 90.46 ± 20.10	LDL-C: I: 92.79 ± 17.78 C: 95.49 ± 20.49	
			HDL-C: I: 59.15 ± 14.69 C: 57.99 ± 15.07	HDL-C: I: 59.53 ± 14.30 C: 61.86 ± 14.30	
			LDL-C/HDL-C: I: 1.56 ± 1.34 C: 1.55 ± 1.33	LDL-C/HDL-C: I: 1.56 ± 1.24 C: 1.54 ± 1.43	
			PLs: I: 2.13 ± 0.36 C: 2.12 ± 0.33	PLs: I: 2.09 ± 0.29 C: 2.09 ± 0.35	
			Lyso-PC: I: 0.074 ± 0.017 C: 0.0069 ± 0.015	Lyso-PC: I: 0.076 ± 0.013 C: 0.072 ± 0.015	
			PC: I: 1.28 ± 0.23 C: 1.28 ± 0.21	PC: I: 1.27 ± 0.18 C: 1.28 ± 0.22	
			PE: I: 0.08 ± 0.03 C: 0.09 ± 0.02	PE: I: 0.10 ± 0.02 C: 0.10 ± 0.02	
			PI: I: 0.09 ± 0.02 C: 0.08 ± 0.02	PI: I: 0.09 ± 0.01 C: 0.08 ± 0.02	
			CRP: I: 1.97 ± 2.65 C: 3.26 ± 6.90	CRP: I: 1.50 ± 1.79 C: 1.43 ± 1.48	
Ota et al., 2015 (40)			Knee extension: I: 34.1 ± 3.2 C: 32.2 ± 3.1	Knee extension: I: 37.1 ± 3.8 C: 33.8 ± 3.2	There were no clinically significant changes in BMI, glucose, blood pressure, serum profile and muscle strength after the dietary SM-rich globular membrane protein consumption.
			MFCV: I: 5.77 ± 0.32 C: 5.91 ± 0.39	MFCV: I: 5.58 ± 0.39 C: 4.17 ± 0.45	
			TC: I: 219 ± 9.8 C: 206 ± 9.2	TC: I: 212 ± 7.5 C: 211 ± 9.9	
			TG: I: 104 ± 9.4 C: 132 ± 12.7	TG: I: 95 ± 8.6 C: 109 ± 10.9	
			Glucose: I: 90.4 ± 2.13 C: 93.3 ± 2.01	Glucose: I: 88.6 ± 1.96 C: 90.4 ± 2.22	
			AST: I: 22.1 ± 0.98 C: 24.5 ± 1.82	AST: I: 22.7 ± 1.06 C: 24.1 ± 1.52	
			ALT: I: 20.1 ± 2.02 C: 25.1 ± 4.33	ALT: I: 19.7 ± 2.38 C: 22.9 ± 2.79	
Minegishi et al., 2016 (49)			BF%: I: 25.2 ± 1.8 C: 25.8 ± 2.1	BF%: I: 25.5 ± 1.6 C: 26.4 ± 2.22	There were no overall changes in BF%, while knee extension strength and MFCV markedly increased after the dietary SM-rich globular membrane protein consumption.
			Knee extension: I: 27.9 ± 2.5 C: 28.2 ± 2.0	Knee extension: I: 32.3 ± 2.6 C: 29.6 ± 2.1	
			MFCV: I: 4.72 ± 0.28 C: 4.65 ± 0.28	MFCV: I: 5.55 ± 0.27 C: 4.62 ± 0.32	
Weiland et al., 2016 (42)			BMI: I: 30.7 ± 1.9 C: 30.9 ± 2.9	BMI: I: 30.4 ± 2.0 C: 30.8 ± 2.8	Consumption of SM-rich milk did not affect plasma lipid parameters.
			BF%: I: 31.2 ± 2.6 C: 30.8 ± 2.4	BF%: I: 30.9 ± 2.8 C: 30.3 ± 2.6	
			SBP: I: 139.6 ± 21.6 C: 131.7 ± 17.2	SBP: I: 135.3 ± 19.1 C: 131.7 ± 16.4	
			DBP: I: 86.4 ± 10.6 C: 82.7 ± 8.9	DBP: I: 84.0 ± 11.0 C: 82.6 ± 8.9	
			TC: I: 233.12 ± 31.70 C: 220.36 ± 36.34	TC: I: 228.10 ± 40.21 C: 221.14 ± 39.82	
			TG: I: 129.36 ± 70.88 C: 124.93 ± 43.41	TG: I: 132.90 ± 56.70 C: 150.62 ± 94.80	
			LDL-C: I: 146.13 ± 31.70 C: 138.40 ± 29.38	LDL-C: I: 148.45 ± 34.41 C: 142.66 ± 31.31	
			HDL-C: I: 55.28 ± 12.76 C: 51.03 ± 13.14	HDL-C: I: 54.90 ± 14.30 C: 50.26 ± 10.82	
			LDL-C/HDL-C: I: 2.64 ± 2.48 C: 2.71 ± 2.23	LDL-C/HDL-C: I: 2.70 ± 2.40 C: 2.84 ± 2.89	
			ApoB: I: 1.28 ± 0.27 C: 1.25 ± 0.23	ApoB: I: 1.27 ± 0.25 C: 1.26 ± 0.22	
			ApoA1: I: 1.64 ± 0.21 C: 1.56 ± 0.24	ApoA1: I: 1.61 ± 0.23 C: 1.56 ± 0.22	
			Glucose: I: 98.82 ± 11.88 C: 99.18 ± 8.46	Glucose: I: 97.56 ± 11.34 C: 96.30 ± 7.74	
			Insulin: I: 14.87 ± 8.78 C: 17.40 ± 8.55	Insulin: I: 15.45 ± 9.07 C: 17.97 ± 16.48	
			HOMA-IR: I: 3.20 ± 2.06 C: 3.74 ± 2.03	HOMA-IR: I: 3.30 ± 2.16 C: 3.74 ± 3.72	
Yoshinaka et al., 2018 (50)			ALT: I: 17.5 ± 9.9 C: 18.0 ± 8.6	ALT: I: 17.3 ± 7.8 C: 20.9 ± 10.5	Ingestion of milk fat globular membrane containing SM had no significant changes on BMI, BF% and knee extension.
			AST: I: 25.0 ± 7.1 C: 26.2 ± 7.3	AST: I: 23.5 ± 6.7 C: 25.1 ± 6.2	
			CRP: I: 2.53 ± 3.03 C: 2.19 ± 2.32	CRP: I: 1.75 ± 1.47 C: 1.42 ± 1.30	
			BMI: I: 21.6 ± 3.1 C: 21.8 ± 2.5	BMI: I: 21.5 ± 3.0 C: 21.7 ± 2.4	
			BF%: I: 26.8 ± 7.5 C: 26.7 ± 6.9	BF%: I: 26.3 ± 7.4 C: 27.4 ± 7.5	
			Knee extension: I: 28.6 ± 9.2 C: 28.8 ± 9.4	Knee extension: I: 27.1 ± 7.8 C: 29.0 ± 8.3	
Vors et al., 2020 (41)			BMI: I: 24 mg/d: 29.05 ± 0.58 65 mg/d: 29.18 ± 0.56 C: 30.22 ± 0.76	BMI: I: 24 mg/d: 29.03 ± 0.57 65 mg/d: 29.06 ± 0.66 C: 30.23 ± 0.83	Consuming SM-rich milk can lower cardiovascular lipid levels, and improve heart health by decreasing several lipid cardiovascular markers.
			SBP: I: 24 mg/d: 124.68 ± 4.17 65 mg/d: 124.47 ± 3.94 C: 124.32 ± 2.74	SBP: I: 24 mg/d: 123.05 ± 6.17 65 mg/d: 120.26 ± 6.82 C:119.53 ± 5.02	
			DBP: I: 24 mg/d: 76.21 ± 2.07 65 mg/d: 75.84 ± 1.94 C: 71.68 ± 2.28	DBP: I: 24 mg/d: 74.37 ± 3.94 65 mg/d: 72.95 ± 3.18 C: 72.52 ± 3.97	
			TC: I: 24 mg/d: 215.72 ± 7.73 65 mg/d: 219.58 ± 9.28 C: 216.11 ± 6.57	TC: I: 24 mg/d: 207.60 ± 11.59 65 mg/d: 204.12 ± 12.75 C: 214.56 ± 10.44	
			TG: I: 24 mg/d: 109.86 ± 9.74 65 mg/d: 130.24 ± 11.52 C: 109.86 ± 7.08	TG: I: 24 mg/d: 108.98 ± 17.72 65 mg/d: 156.82 ± 7.08 C: 119.61 ± 12.40	
			LDL-C: I: 24 mg/d: 136.86 ± 6.57 65 mg/d: 140.33 ± 7.34 C: 143.04 ± 5.80	LDL-C: I: 24 mg/d: 130.28 ± 9.66 65 mg/d: 127.19 ± 10.44 C: 141.49 ± 8.89	
			HDL-C: I: 24 mg/d: 47.55 ± 1.93 65 mg/d: 45.23 ± 2.32 C: 44.85 ± 1.93	HDL-C: I: 24 mg/d: 46.01 ± 3.09 65 mg/d: 47.55 ± 3.09 C: 44.07 ± 3.09	
			LDL-C/HDL-C: I: 24 mg/d: 2.88 ± 3.40 65 mg/d: 3.10 ± 3.16 C: 3.18 ± 3.00	LDL-C/HDL-C: I: 24 mg/d: 2.83 ± 3.12 65 mg/d: 2.67 ± 3.38 C: 3.21 ± 2.88	
			ApoB: I: 24 mg/d: 1.01 ± 0.05 65 mg/d: 1.02 ± 0.06 C: 24 mg/d 1.03 ± 0.05	ApoB: I: 24 mg/d: 0.07 ± 0.07 65 mg/d: 0.03 ± 0.09 C: 1.04 ± 0.07	
			ApoA1: I: 24 mg/d: 1.17 ± 0.02 65 mg/d: 1.19 ± 0.03 C: 1.16 ± 0.02	ApoA1: I: 24 mg/d: 1.17 ± 0.04 65 mg/d: 1.18 ± 0.05 C: 1.15 ± 0.04	
			Glucose: I: 24 mg/d: 91.98 ± 1.98 65 mg/d: 92.70 ± 1.8 C: 94.14 ± 1.8	Glucose: I: 24 mg/d: 92.34 ± 3.06 65 mg/d: 90.72 ± 2.88 C: 93.60 ± 3.06	
			Insulin: I: 24 mg/d: 7.11 ± 0.72 65 mg/d: 8.07 ± 1.36 C:7.29 ± 0.99	Insulin: I: 24 mg/d: 7.05 ± 1.33 65 mg/d: 7.66 ± 2.26 C: 7.72 ± 1.62	
			HOMA-IR: I: 24 mg/d: 1.67 ± 0.19 65 mg/d: 1.82 ± 0.28 C: mg/d: 1.74 ± 0.26	HOMA-IR: I: 24 mg/d: 1.64 ± 0.33 65 mg/d: 1.72 ± 0.46 C: 1.91 ± 0.43	
Barz et al., 2021 (48)	I: 750 mg/d: 0.43 ± 0.015 1,250 mg/d: 0.422 ± 0.018C: 0.436 ± 0.017	I: 750 mg/d: 0.413 ± 0.027 1,250 mg/d: 0.395 ± 0.027C: 0.407 ± 0.029	PLs: I: 750 mg/d: 2.20 ± 0.05	PLs: I: 750 mg/d: 2.14 ± 0.10	Supplementation with SM-rich milk was associated with a reduction in atherogenic SMand Cer species, which improved cardiovascular risk markers.
			1,250 mg/d: 2.30 ± 0.06 C: 2.28 ± 0.09	1,250 mg/d: 2.12 ± 0.10 C: 2.21 ± 0.14	
			Lyso-PC: I: 750 mg/d: 0.0899 ± 0.047 1,250 mg/d: 0.0938 ± 0.048 C: 0.0957 ± 0.063	Lyso-PC: I: 750 mg/d: 0.089 ± 0.080 1,250 mg/d: 0.086 ± 0.082 C: 0.0925 ± 0.010	
			PC: I: 750 mg/d: 1.44 ± 0.04 1,250 mg/d: 1.54 ± 0.04 C: 1.50 ± 0.05	PC: I: 750 mg/d: 1.40 ± 0.07 1,250 mg/d: 1.42 ± 0.07 C: 1.48 ± 0.09	
			PE: I: 750 mg/d: 0.052 ± 0.0031 1,250 mg/d: 0.054 ± 0.0028 C: 0.049 ± 0.0036	PE: I: 750 mg/d: 0.048 ± 0.0059 1,250 mg/d: 0.052 ± 0.0047 C: 0.048 ± 0.0052	
			PI: I: 750 mg/d: 0.195 ± 0.016 1,250 mg/d: 0.188 ± 0.016 C: 0.192 ± 0.019	PI: I: 750 mg/d: 0.18 ± 0.029 1,250 mg/d: 0. 17 ± 0.032 C: 0.181 ± 0.031	

No, number of participants; I, intervention group; C, control group; Outcome Indicators: SBP, systolic blood pressure; DBP, diastolic blood pressure; BMI, body mass index; BF%, body fat percentage; Knee extension; MFCV, muscle fiber conduction velocity; SM, sphingomyelin; PLs, phospholipids; Lyso-PC, Lyso-phosphatidyl cholines; PC, phosphatidyl cholines; PE, phosphatidyl-ethanolamine; PI, phosphatidyl-inositol; TC, total cholesterol; TG, triglyceride; LDL-C, low-density lipoprotein cholesterol; HDL-C, high-density lipoprotein cholesterol; LDL-C/HDL-C; Glucose; Insulin; HOMA-IR, homeostasis model assessment of insulin resistance; AST, aspartate transaminase; ALT, alanine transaminease; CRP, C-reactive protein.

### 3.4 Results of the meta-analyses

#### 3.4.1 Impact of dietary SM on blood SM level

**Figure 3:**
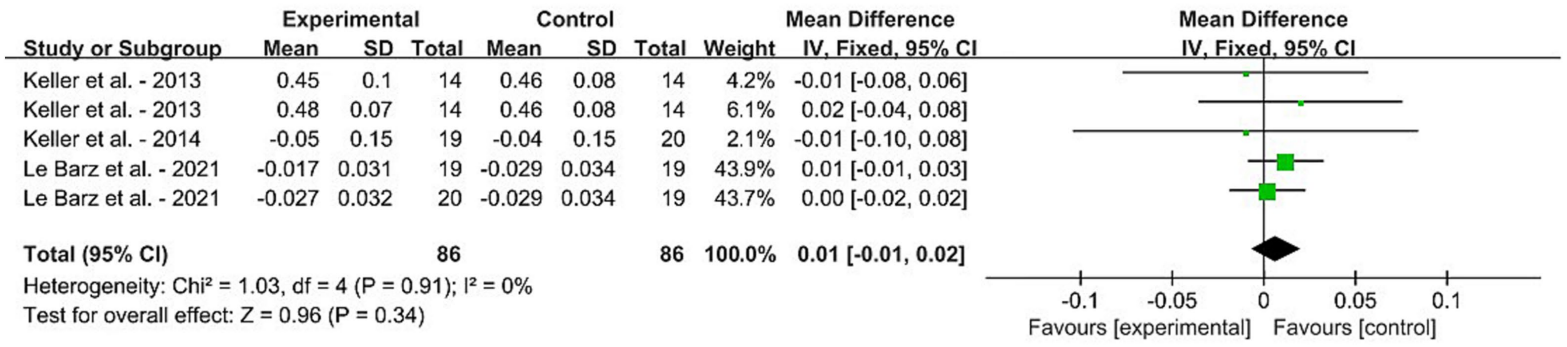
Forest plots for the effects of sphingomyelin (SM) on blood SM in adults without metabolic syndrome (MetS). The horizontal bar represents the 95% confidence interval (CI). The magnitude of the rectangle at the center of the horizontal bar is proportional to the weight of the provided study. As indicated by the diamond at the bottom, the pooled mean difference (MD) is present. **(A)** BMI, **(B)** BF%, **(C)** knee extension, **(D)** MFCV, **(E)** SBP, and **(F)** DBP.

For the daily dietary SM intervention-based meta-analysis, Figure 3 displays the forest plot results for the MD in serum SM levels among adults without MetS from five RCTs. Squares represent the MD for RCTs, while line segments crossing the squares, which are aligned parallel to the X-axis, depict the 95% CIs. The pooled MD is represented by diamonds indicating the effect size and confidence interval of multiple studies combined. The result showed that the dietary SM intervention did not affect the blood SM level in healthy adults [MD: 0.01, 95% CI (−0.01, 0.02), *p* = 0.34].

#### 3.4.2 Impact of dietary SM on anthropometric parameters and blood pressure

**Figure 4:**
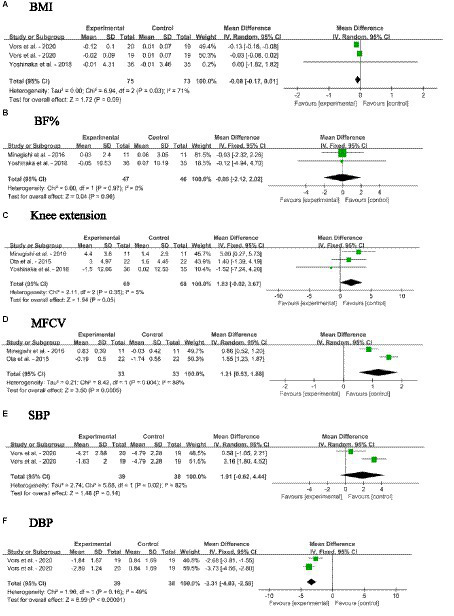
Forest plots for the effects of sphingomyelin (SM) on anthropometric parameters and blood pressure in adults without metabolic syndrome (MetS). **(A)** BMI, body mass index; **(B)** BF%, body fat percentage; **(C)** Knee extension; **(D)** MFCV, muscle fiber conduction velocity; **(E)** SBP, systolic blood pressure; **(F)** DBP, diastolic blood pressure. The horizontal bar represents the 95% confidence interval (CI). The magnitude of the rectangle at the center of the horizontal bar is proportional to the weight of the provided study. As indicated by the diamond at the bottom, the pooled mean difference (MD) is present.

The effects of SM consumption on anthropometric parameters and blood pressure in adults without MetS are illustrated in Figures 4A–F. These parameters include BMI, BF%, knee extension, MFCV, SBP and DBP. According to the findings, SM consumption significantly decreased DBP [MD: −3.31, 95% CI (−4.03, −2.58), *p* < 0.00001], while significantly increased MFCV [MD: 1.21, 95% CI (0.53, 1.88), *p* = 0.0005] when compared with the control group. It is noteworthy to mention that the DBP remained within the range of 70–80 mmHg even after the fall. There were no significant changes in BMI, BF%, knee extension and SBP.

#### 3.4.3 Impact of dietary SM on blood lipid profile

**Figure 5:**
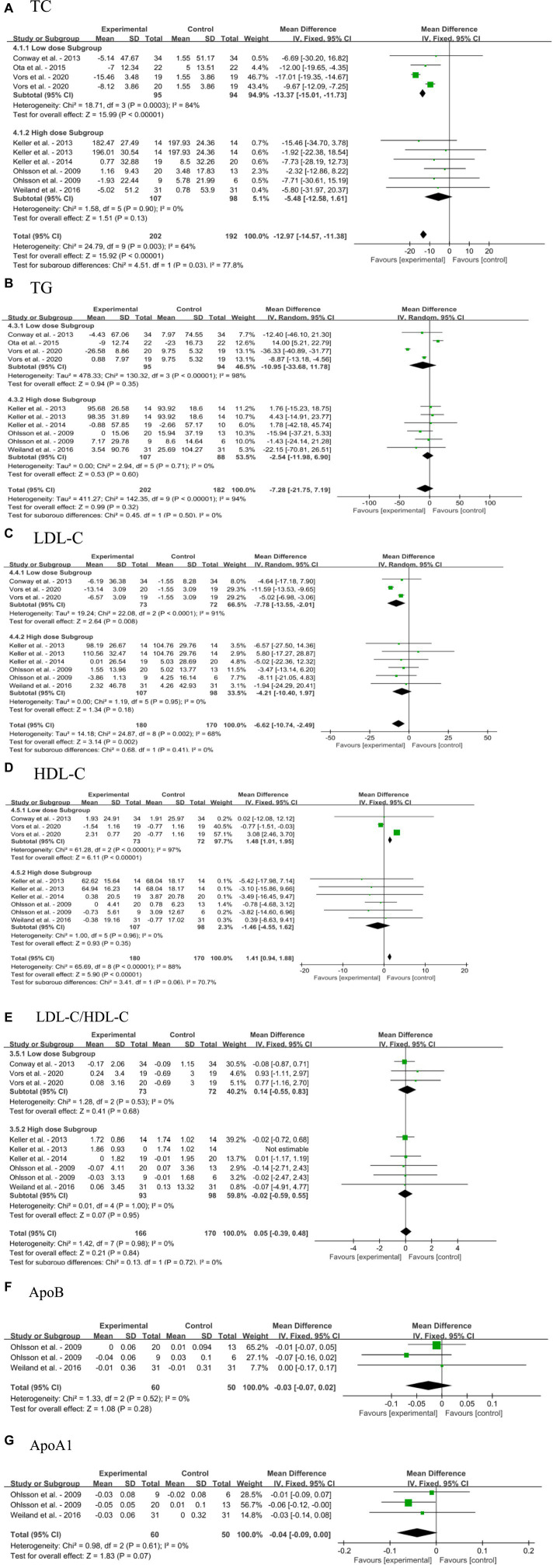
Forest plots for the effects of sphingomyelin (SM) on blood lipid profile in adults without metabolic syndrome (MetS). **(A)** TC, total cholesterol; **(B)** TG, triglyceride; **(C)** LDL-C, low-density lipoprotein cholesterol; **(D)** HDL-C, high-density lipoprotein cholesterol; **(E)** LDL-C/HDL-C; **(F)** ApoB, apolipoprotein B; **(G)** ApoA1, apolipoprotein A1. The horizontal bar represents the 95% confidence interval (CI). The magnitude of the rectangle at the center of the horizontal bar is proportional to the weight of the provided study. As indicated by the diamond at the bottom, the pooled mean difference (MD) is present.

Overall, dietary SM had little effect on blood lipid profile in adults without MetS, including TC, TG, LDL-C, HDL-C, LDL-C/HDL-C, ApoB, and ApoA1, as shown in Figures 5A–G. The overall effects of dietary SM supplementation significantly decreased the blood TC [MD: −12.97, 95% CI: (−14.57, −11.38), *p* < 0.00001] and LDL-C [MD: −6.62, 95% CI: (−10.74, −2.49), *p* = 0.002] levels, and increased HDL-C level [MD:1.41, 95% CI: (0.94, 1.88), *p* < 0.00001]. Upon examining various concentrations, the meta-analysis results indicated that low dose dietary SM intervention (≤ 400 mg/day) significantly decreased in blood TC [MD: −13.37, 95% CI: (−15.01, −11.73), *p* < 0.00001] and LDL-C [MD: −7.78, 95% CI: (−13.55, −2.01), *p* = 0.008] levels, increased in HDL-C [MD:1.48, 95% CI: (1.01, 1.95), *p* < 0.00001] level, while the other blood lipid indicators were not affected. However, the high dose dietary SM treatment did not change in any parameter of the blood lipid profile.

#### 3.4.4 Impact of dietary SM on blood phospholipid profile

**Figure 6:**
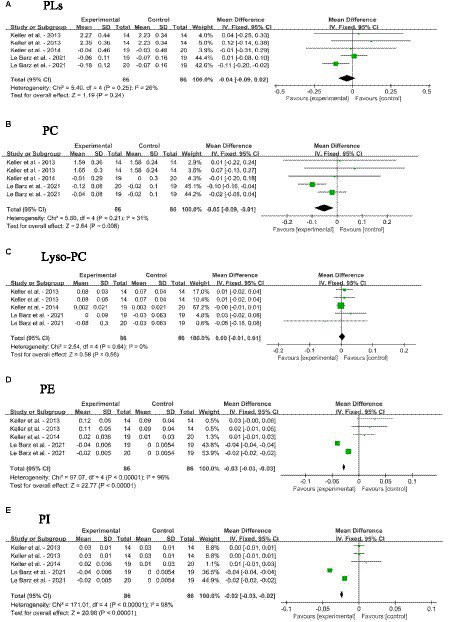
Forest plots for the effects of sphingomyelin (SM) on blood phospholipid levels in adults without metabolic syndrome (MetS). **(A)** PLs, total phospholipids; **(B)** PC, phosphatidyl cholines; **(C)** Lyso-PC, Lyso-phosphatidyl cholines; **(D)** PE, phosphatidyl-ethanolamine; **(E)** PI, phosphatidyl-inositol. The horizontal bar represents the 95% confidence interval (CI). The magnitude of the rectangle at the center of the horizontal bar is proportional to the weight of the provided study. As indicated by the diamond at the bottom, the pooled mean difference (MD) is present.

The impacts of dietary SM supplementation on blood PLs, PC, lyso-PC, PE, and PI levels in adults without MetS are depicted in Figures 6A–E. Dietary SM intervention did not change the levels of PLs and Lyso-PC. The intake of SM slightly decreased the blood PC [MD: −0.05; 95% CI (−0.09, −0.01), *p* = 0.008], PE [MD: −0.03; 95% CI (−0.03, −0.03), *p* < 0.0001] and PI [MD: −0.02; 95% CI (−0.03, −0.02), *p* < 0.0001] levels.

#### 3.4.5 Impact of dietary SM on blood glycemic indices

**Figure 7:**
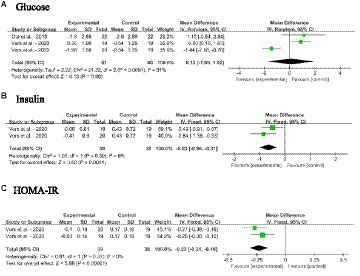
Forest plots for the effects of sphingomyelin (SM) on blood glycemia aspect in adults without metabolic syndrome (MetS). **(A)** Glucose, **(B)** insulin, **(C)** HOMA-IR, homeostasis model assessment of insulin resistance. The horizontal bar represents the 95% confidence interval (CI). The magnitude of the rectangle at the center of the horizontal bar is proportional to the weight of the provided study. As indicated by the diamond at the bottom, the pooled mean difference (MD) is present.

Figures 7A–C shows the meta-analysis results of blood glucose, insulin and HOMA-IR in adults without MetS after dietary SM supplementation. The dietary SM supplementation did not affect the glucose level. On the other hand, SM intervention significantly decreased the insulin level [MD: −0.63; 95% CI (−0.96, −0.31), *p* = 0.0001] and HOMA-IR [MD: −0.23; 95% CI (−0.31, −0.16), *p* < 0.00001].

#### 3.4.6 Impact of dietary SM on liver function biomarkers

**Figure 8:**
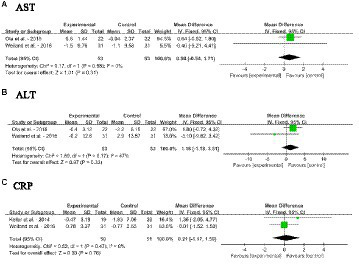
Forest plots for the effects of sphingomyelin (SM) on liver function biomarkers in adults without metabolic syndrome (MetS). **(A)** AST, aspartate transaminase; **(B)** ALT, alanine transaminease; **(C)** CRP, C-reactive protein. The horizontal bar represents the 95% confidence interval (CI). The magnitude of the rectangle at the center of the horizontal bar is proportional to the weight of the provided study. As indicated by the diamond at the bottom, the pooled mean difference (MD) is present.

Forest plots for the effects of dietary SM intervention on liver function biomarkers, AST, ALT and CRP in adults without MetS are shown in Figures 8A–C. The dietary SM supplementation did not significantly affect serum AST, ALT, and CRP levels.

### 3.5 Publication bias

**Figure 9:**
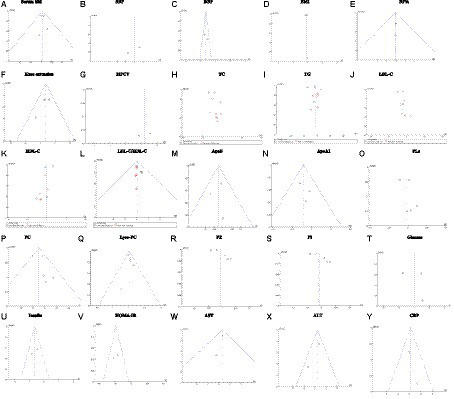
Sensitivity analysis plot. **(A)** Serum SM, Serum sphingomyelin; **(B)** SBP, systolic blood pressure; **(C)** DBP, diastolic blood pressure; **(D)** BMI, body mass index, **(E)** BF%, body fat percentage; **(F)** Knee extension; **(G)** MFCV, muscle fiber conduction velocity; **(H)** TC, total cholesterol; **(I)** TG, triglyceride; **(J)** LDL-C, low-density lipoprotein cholesterol; **(K)** HDL-C, high-density lipoprotein cholesterol; **(L)** LDL-C/HDL-C, **(M)** ApoA, apolipoprotein A; **(N)** ApoB, apolipoprotein B; **(O)** PLs, phospholipids; **(P)** Lyso-PC, Lyso-phosphatidyl choline; **(Q)** PC, phosphatidyl choline; **(R)** PE, Phosphatidyl-ethanolamine; **(S)** PI, Phosphatidyl-inositol; **(T)** glucose; **(U)** insulin; **(V)** HOMA-IR, homeostasis model assessment of insulin resistance; **(W)** AST, aspartate transaminase; **(X)** ALT, alanine transaminease; **(Y)** CRP, C-reactive protein. Fixed effects model: **(A,C,E,F,L–N,P,Q,U–Y)**. Random effects model: **(B,D,G–K,O,R–T)**.

Publication bias was assessed using funnel plots as shown in Figure 9. Funnel plots for serum SM, BF%, knee extension, MFCV, SBP, DBP, TC, TG, LDL-C, HDL-C, LDL-C/HDL-C, ApoB, ApoA1, PL, PC, Lyso-PC, PE, PI, glucose, insulin, HOMA-IR, AST, ALT, CRP are shown, and publication bias may be present. However, due to the small number of literatures included, the results of funnel plot may not be sufficient to fully prove publication bias.

## 4 Discussion

SM is found in the brain, plasma, skin, as well as in dietary sources such as dairy products, meat, eggs, aquatic products and soybeans (10). Sphingolipid metabolites are regarded as bioactive lipids and are involved in numerous crucial cellular processes, including cell survival, apoptosis, metabolism, immune cell trafficking, autophagy, and mitochondrial function (51). SM and its metabolites Cer, C1P, and S1P are involved in MetS-related pathophysiological processes, including CVDs, T2DM, inflammation, non-alcoholic fatty disease, and cancer (52). Whether exogenous SM positively or negatively affects human health deserved to be evaluated thoroughly. Our systemic review and meta-analyze of the RCT data first systematically examined the effects of dietary SM on metabolic indexes in adults according to PRISMA principles. By employing explicit data extraction techniques and conducting rigorous and comprehensive searches across multiple databases, this meta-analysis is distinguished by its comprehensive indicators in adults.

Our meta-analysis of RCTs showed that dietary SM supplementation did not affect the blood PLs and SM levels, as well as anthropometric markers including BMI and BF% in adults without MetS. Meanwhile, the dietary SM supplementation increased MFCV and improved neuromuscular functions in adults. As reported by Le Barz et al., the intervention group that received 750 or 1,250 mg/d dietary SM from milk showed no significant differences in the concentrations of serum SM, Cer and PLs when compared with the control group (48). One possible reason for this is that the majority of SM and its hydrolysate Cer could not be absorbed in their original form and hence, do not contribute to the chylomicron and plasma SM pools (33, 48). An early study indicated that approximately 40% of ingested SM might be excreted intact as SM, Cer or sphingosine in the feces (53). Large amounts of dietary SM remain in the intestine unabsorbed due to absorption barriers in adults, which is likely co-excreted with cholesterol (54). Dietary SM and its metabolites have been shown to inhibit cholesterol absorption in Caco-2 cells and animal studies (28–30), and substantially alter the metabolism of TG and TC in rats (31, 32). SM possesses a greater interface area and a more effective capacity for hydrogen bond formation in comparison to other PLs, which is critical for facilitating interactions between SM and other lipids within the cell membrane (55). The crucial intermolecular hydrogen bond is established through the interaction between the hydroxyl group of cholesterol and the 2-NH of SM. Additionally, the formation of intermolecular hydrogen bonds and a network by the phosphate oxygen and 3-OH between SM molecules may impede the release of cholesterol from these lipid complexes (56). By virtue of their strong affinity for cholesterol, SM impedes the transfer of micellar lipids to the enterocytes, and inhibits luminal hydrolysis and micellar solubilization (57). A four-week milk SM intervention resulted in a significant increase in fecal SM and Cer in comparison to the control group (48). It is unknown whether this is the result of decreased SM synthesis in enterocytes or liver following dietary SM intervention. Alternatively, the size of the body’s total SM pool is large enough that the absorbed amount of dietary SM is an insignificant fraction of it. Whether any of these hypotheses is true remains to be determined.

The basic and clinical researchers agree that milk SM provides protection against dysfunctional lipid metabolism, intestinal dysbiosis, and inflammation (55). It is noteworthy that the inhibitory effect of SM derived from milk on cholesterol absorption surpasses that of SM derived from eggs. This disparity may be attributed to the presence of longer chain and greater degree of saturation of the fatty acyl group in the milk SM than that in the egg ones (55, 58). Milk SM has substantial proportion of very-long chain fatty acids (C22:0-C24:0) and diverse sphingoid bases (d16:0 to d19:0) (34, 57). Dietary SM is hydrolyzed in the intestinal mucosa by alkaline sphingomyelinase to Cer, and phosphorylcholine. Cer is subsequently hydrolyzed by neutral ceramidase and bile salt-stimulated lipase into sphingosine and fatty acids, which are then absorbed into the enterocytes. The majority of sphingosine is either dephosphorylated to generate S1P by sphingosine kinase, or to a lesser degree, form new Cer and SM (10). Multiple studies have demonstrated that distinct cellular functions can be carried out by various Cer species, contingent upon the fatty acyl chains that they contain (59). During the progression of T2DM and NAFLD, C16-Cer and C18-Cer species are thought to be generated *de novo* and deleterious in the liver and muscle tissues (60–63). On the contrary, Cer denoted as C22:0-, C24:1, and C24:0- are more widely regarded as having beneficial and protective effects on cells, particularly in the liver (62, 64–66). Research has demonstrated that mice with a deficiency in the Cer synthase 2, which is accountable for producing C24:1, C22:0-, and C24:0-Cer, were more susceptible to diet-induced hepatic steatohepatitis and developed insulin resistance (62). Le Barz et al. discovered that the atherogenic C16-SM, C18-SM, and C24:1-Cer species in serum were significantly reduced by the milk SM intervention, which leads to significant increases in the proportions of C22:0-SM, C24:0-SM, C22:0-Cer, and C24:0-Cer species (48). This might be attributed to the beneficial effects of milk SM.

According to Ramprasath et al., with the exception of an elevated HDL-C concentration, the blood lipid profile in humans remains unaffected by the consumption of 1 g/day of dietary SM. Additionally, cholesterol absorption, synthesis, and intraluminal solubilization remain unaffected when compared to the control group (38). However, our meta-analysis results showed that a low dose of dietary SM intervention (≤ 400 mg/day) significantly reduced the blood TC and LDL-C levels, and increased HDL-C level. The levels of TG, LDL-C/HDL-C, ApoB, and ApoA1 were not changed. Interestingly, the high dose dietary SM treatment did not change the lipid profile. Animal experiments have shown that dietary SM supplementation has no effect on atherosclerosis or circulating SM levels in HFD apoE^−/−^ mice, but it inhibits atherosclerosis in chow-fed apoE^−/−^ mice (27). It is possible that the effects of dietary SM derived from milk on the lipid profile depends on other components in the diet. Alternatively, there is an interaction between the dietary SM and dietary energy content (37). The lack of response in the high dietary SM group could be due to a mechanism that induces excretion of SM in the feces or blocks absorptions of sphingosine when more SM is present in the gastrointestine tract. Whether this mechanism exists remains to be tested.

Circulating Cer has been linked to the development of atherosclerosis and CVDs over the past decade (67). The conversion of SM in the blood LDL-C particles to Cer by sphingomyelinase facilitates Cer aggregation. The increases in SM levels were associated with an early onset of atherosclerosis (68–70). Cer may undergo phosphorylation to form C1P, which promotes inflammation via oxidative stress or tumor necrosis factor-α and causes apoptosis and non-alcoholic steatohepatitis (71, 72). Additionally, hydrolysis of Cer can produce sphingosine, which can be phosphorylated to produce S1P (10). Conversely, S1P has been thought to prevent apoptosis and linked to cellular proliferation and growth (73). The elevated concentration of S1P in the bloodstream serves vital homeostatic roles in preserving the integrity of blood vessels, while the S1P gradient is indispensable for the movement of immune cells (51). Research has indicated that impairments of fatty acid oxidation can be attributed to disturbances in sphingolipid metabolism, where Cer and S1P may play a role (74). The dogma is that accumulation of Cer is detrimental, whereas accumulation of S1P is advantageous (74, 75). So far, there is no discernible impact of dietary SM on blood SM and Cer levels. Therefore, more clinical studies are required to monitor its effects on the endogenous S1P and sphingolipidome in the bloodstream and along the gastrointestinal tract in humans.

Another thing worth noting in this meta-analysis is that dietary SM supplementation reduced insulin levels and HOMA-IR, but did not affect blood glucose or inflammatory response factors. This might be related to the fact that dietary SM does not significantly affect serum SM, Cer, and PL concentrations (48). Congestive complications such as retinopathy, neuropathy, stroke, myocardial infarction, and arteritis of the lower limbs are all possible outcomes of T2DM (76). Studies have shown significant associations between increased levels of circulating Cer and insulin resistance as well as T2DM (25, 77–80). Jensen et al. discovered a positive link between elevated blood levels of Cer and C16-lactosyl-Cer and increased glucose levels. However, no significant associations were detected between SM and glucose levels (80). An intriguing study also shows that diabetic patients had higher circulating Cer levels than nondiabetic individuals (77). In contrast to blood Cer, which is found in conjunction with LDL and very-low-density lipoprotein (VLDL), plasma S1P is bound to both albumin and apolipoprotein M, which associates preferentially with HDL (75). In addition to elevated levels of LDL-Cer, obesity and diabetes are correlated with reduced HDL-S1P levels. It has been demonstrated that LDL-Cer inhibits insulin signaling in muscle, while HDL-S1P improves insulin signaling and enhances the function and survival of pancreatic cells (77, 81, 82). HDL-S1P and LDL-Cer exhibit contrasting effects on the progression of T2DM (76). Hence, the process of interconversion among these sphingolipid species is subject to strict regulation. Even a minor disruption in anabolism, catabolism, or substrate accessibility can result in the atypical accumulation of one or more sphingolipid species, thereby causing an imbalanced supply of fatty acids within the metabolic system (83). As a result, changes in the profiles and concentrations of sphingolipids have emerged as a crucial area of study in MetS research. It is very interesting to find that dietary SM supplementation can reduce blood insulin level. Whether this can be attributed to the abundant long-chain fatty acyl groups in Cer derived from milk SM remains to be analyzed. In addition, whether this reduced insulin level is due to the reduction of insulin secretion or an increase in insulin clearance is also deserved to be investigated.

Nevertheless, the current meta-analysis has a few limitations. Due to the limited number of articles included, the number of participants may not have been sufficient to create a relatively large sample size, thereby increasing the type-2 statistical error. The absence of waist circumference data for anthropometric parameters is the second issue of this study. Furthermore, in some of the included RCTs, the intervention group was given a diet or beverage that various doses of SM supplementation were based on normal intakes. A comprehensive stratified analysis of the blood lipid profile was conducted. However, the dose–response relationship between supplementation and biomarkers including anthropometric parameters, glycemia aspect, and inflammatory response factors could not be determined. Therefore, further comprehensive prospective investigations are required to examine the impact of dietary SM supplementation on the sphingolipidome and Cer-S1P in the gastrointestinal tract and bloodstream. Finally, sample size, intervention period, or other factors in this meta-analysis may also be source of heterogeneity, and stratified analyzes of these factors are also necessary.

## 5 Conclusion

In summary, dietary SM supplementation did not have a detrimental effect on metabolic indexes in adults without MetS, but had a protective effect on blood lipid profiles and insulin level. From the analysis results of the forest map, dietary SM supplementation can improve DBP, levels of TC, LDL-C, HDL-C, and insulin, and HOMA-IR. However, it does not affect BMI, SBP, and levels of TG, LDL-C/HDL-C and glucose. Therefore, dietary SM may serve as a protective factor for MetS, which needs to be confirmed via further clinical trials and basic research.

## Data availability statement

The original contributions presented in the study are included in the article/Supplementary material, further inquiries can be directed to the corresponding authors.

## Author contributions

C-ZL: Conceptualization, Data curation, Investigation, Software, Validation, Writing – original draft. L-MW: Conceptualization, Data curation, Formal analysis, Investigation, Validation, Writing – original draft. C-XZ: Data curation, Investigation, Methodology, Software, Writing – original draft. H-YD: Conceptualization, Data curation, Investigation, Software, Writing – original draft. G-XC: Data curation, Project administration, Supervision, Validation, Writing – review & editing. FY: Conceptualization, Data curation, Funding acquisition, Methodology, Resources, Supervision, Validation, Writing – original draft, Writing – review & editing.

## Footnotes

^1^ https://gradepro.org/
