# Supplementary material for: The impacts of dietary sphingomyelin supplementation on metabolic parameters of healthy adults: a systematic review and meta-analysis of randomized controlled trials
Source: Front Nutr. 2024 Feb 23;11:1363077. doi: 10.3389/fnut.2024.1363077 (PMC10922005; doi:10.3389/fnut.2024.1363077)
Supplement: Supplementary file 1 [file Data_Sheet_1.PDF]

Supplement 1

Pubmed

	Searches	Results
#1	Search: ((((((Sphingomyelin[Title/Abstract]) OR (Sphingolipid[Title/Abstract])) OR (Ceramide[Title/Abstract])) OR (Phospholipid[Title/Abstract])) OR (Phosphatides[Title/Abstract])) OR (Cholesterol[Title/Abstract])) OR (Sphingosine[Title/Abstract]) Filters: Randomized Controlled Trial, Clinical Trial	**23056**
#2	Search: (((milk polar lipids[Title/Abstract])) OR (lipids[Title/Abstract])) AND (adults[Title/Abstract]) Filters: Randomized Controlled Trial, Clinical Trial	**780**
#3	#1 AND #2	**429**
#4	Search: (((((((((Sphingomyelin) OR (Sphingolipid)) OR (Ceramide)) OR (Phospholipid)) OR (Phosphatides)) OR (Cholesterol)) OR (Sphingosine) AND (randomized controlled trial[Filter])) AND ((((((milk polar lipids)) OR (lipids)) OR (adults) AND (randomized controlled trial[Filter])) Filters: Randomized Controlled Trial, in the last 20 years	**357**

Web of Science

	searches	results
#1	((((TI=(Milk-based phospholipids)) OR TI=(Milk polar lipids)) O R TI=(Sphingolipids)) OR TI=(Lipids)) AND TI=(adults)	2258
#2	(((((TI=(sphingomyelin)) OR TI=(Sphingolipid)) OR TI=(ceramide)) OR TI=(Phospholipid)) OR TI=(Phosphatides)) OR TI=(sphingosine)	43891
#3	#1 AND #2	7
#4	TI=(randomized controlled trial)	16584 7

#5	#3 AND #4	0

The Cochrane Library

ID Search Hits

#1 (Sphingomyelin): ti, ab, kw 184

#2 (Ceramide): ti, ab, kw 312

#3 (sphingosine): ti, ab, kw 521

#4 (Phospholipid): ti, ab, kw 1562

#5 (Cholesterol): ti, ab, kw 43100

#6 (Sphingolipids): ti, ab, kw 108

#7 (#1 or #2 or #3 or #4 or #5 or #6 AND "randomized-controlled trials"): ti, ab, kw 504

#8 (Milk-based phospholipids): ti, ab, kw 5

#9 (Milk polar lipids): ti, ab, kw 16

#10 #8 or #9 AND "randomized-controlled trials" 479

#11 #7 AND #10 AND "randomized-controlled trials" 16856

Embase

#1 sphingomyelin: kw 1691

#2 'milk based' AND phospholipids: kw 4

#3 milk AND polar AND lipids: kw 49

#4 sphingolipids: kw 2231

#5 #1or #2or #3 or#4 3853

#6 #5 and randomized-controlled trials" 33

Clinical Trails

#1 Intervention/treatment（sphingomyelin）: 9

#2 Intervention/treatment（milk polar lipids）:7

#3 Intervention/treatment（phospholipid）:251

#4 Intervention/treatment (lipids) :6215

Supplement 2

Author(s):
Question: Serum SM compared to placebo for [health problem]
Setting:
Bibliography: . [Intervention] for [health problem]. Cochrane Database of Systematic Reviews [Year], Issue [Issue].

Certainty assessment							№ of patients		Effect		Certainty	Importance
№ of studies	Study design	Risk of bias	Inconsistency	Indirectness	Imprecision	Other considerations	Serum SM	placebo	Relative (95% CI)	Absolute (95% CI)		
**Serum SM**												
3	randomised trials	not serious	not serious	not serious	not serious	none	72	72	-	MD **0.01 higher** (0.01 lower to 0.02 higher)	⊕⊕⊕⊕ High	

CI: confidence interval; **MD:** mean difference

Author(s):
Question: Anthropometric parameters compared to placebo for [health problem]
Setting:
Bibliography: . [Intervention] for [health problem]. Cochrane Database of Systematic Reviews [Year], Issue [Issue].

Certainty assessment							№ of patients		Effect		Certainty	Importance
№ of studies	Study design	Risk of bias	Inconsistency	Indirectness	Imprecision	Other considerations	Anthropometric parameters	placebo	Relative (95% CI)	Absolute (95% CI)		
**BMI**												
2	randomised trials	not serious	serious	not serious	not serious	none	75	73	-	MD **0.08 lower** (0.17 lower to 0.01 higher)	⊕⊕⊕◯ Moderate	
**BF%**												
2	randomised trials	not serious	not serious	not serious	not serious	none	47	46	-	MD **0.05 lower** (2.12 lower to 2.02 higher)	⊕⊕⊕⊕ High	
**Knee extension**												
3	randomised trials	not serious	not serious	not serious	not serious	none	69	68	-	MD **1.83 higher** (0.02 lower to 3.67 higher)	⊕⊕⊕⊕ High	
**MFCV**												
2	randomised trials	not serious	serious	not serious	not serious	none	33	33	-	MD **1.21 higher** (0.53 higher to 1.88 higher)	⊕⊕⊕◯ Moderate	
**SBP**												
1	randomised trials	not serious	serious	not serious	not serious	none	39	38	-	MD **1.91 higher** (0.62 lower to 4.44 higher)	⊕⊕⊕◯ Moderate	
**DBP**												
1	randomised trials	not serious	not serious	not serious	not serious	none	39	38	-	MD **3.31 lower** (4.03 lower to 2.58 lower)	⊕⊕⊕⊕ High	

CI: confidence interval; **MD:** mean difference

Author(s):
Question: Serum lipid compared to placebo for [health problem]
Setting:
Bibliography: . [Intervention] for [health problem]. Cochrane Database of Systematic Reviews [Year], Issue [Issue].

Certainty assessment							№ of patients		Effect		Certainty	Importance
№ of studies	Study design	Risk of bias	Inconsistency	Indirectness	Imprecision	Other considerations	Serum lipid	placebo	Relative (95% CI)	Absolute (95% CI)		
**TC**												
7	randomised trials	not serious	serious	not serious	not serious	none	188	178	-	MD **10.1 lower** (14.93 lower to 5.27 lower)	⨁⨁⨁◯ Moderate	
**TC - Low dose Subgroup**												
3	randomised trials	not serious	serious	not serious	not serious	none	95	94	-	MD **12.68 lower** (18.28 lower to 7.07 lower)	⨁⨁⨁◯ Moderate	
**TC - High dose Subgroup**												
4	randomised trials	not serious	not serious	not serious	not serious	none	93	84	-	MD **2.83 lower** (10.73 lower to 5.07 higher)	⨁⨁⨁⨁ High	
**TG**												
7	randomised trials	not serious	serious	not serious	not serious	none	188	168	-	MD **9.08 lower** (24.46 lower to 6.29 higher)	⨁⨁⨁◯ Moderate	
**TG - Low dose Subgroup**												
3	randomised trials	not serious	serious	not serious	not serious	none	95	94	-	MD **11.44 lower** (33.67 lower to 10.78 higher)	⨁⨁⨁◯ Moderate	
**TG - High dose Subgroup**												
4	randomised trials	not serious	serious	not serious	not serious	none	93	74	-	MD **6.43 lower** (18.74 lower to 5.88 higher)	⨁⨁⨁◯ Moderate	
**LDL-C**												
6	randomised trials	not serious	serious	not serious	not serious	none	166	156	-	MD **6.6 lower** (10.95 lower to 2.25 lower)	⨁⨁⨁◯ Moderate	
**LDL-C - Low dose Subgroup**												
2	randomised trials	not serious	serious	not serious	not serious	none	73	72	-	MD **7.78 lower** (13.55 lower to 2.01 lower)	⨁⨁⨁◯ Moderate	
**LDL-C - High dose Subgroup**												
4	randomised trials	not serious	not serious	not serious	not serious	none	93	84	-	MD **4.06 lower** (10.64 lower to 2.53 higher)	⨁⨁⨁⨁ High	
**HDL-C**												
6	randomised trials	not serious	serious	not serious	not serious	none	166	156	-	MD **0.29 higher** (2.43 lower to 3.01 higher)	⨁⨁⨁◯ Moderate	
**HDL-C - Low dose Subgroup**												
2	randomised trials	not serious	serious	not serious	not serious	none	73	72	-	MD **1.08 higher** (2.52 lower to 4.67 higher)	⨁⨁⨁◯ Moderate	

**HDL-C - High dose Subgroup**												
4	randomised trials	not serious	not serious	not serious	not serious	none	93	84	-	MD **0.94 lower** (4.16 lower to 2.28 higher)	⊕⊕⊕⊕ High	
**LDL-C/HDL-C**												
6	randomised trials	not serious	not serious	not serious	not serious	none	166	156	-	MD **0.1 higher** (0.39 lower to 0.59 higher)	⊕⊕⊕⊕ High	
**LDL-C/HDL-C - Low dose Subgroup**												
2	randomised trials	not serious	not serious	not serious	not serious	none	73	72	-	MD **0.14 higher** (0.55 lower to 0.83 higher)	⊕⊕⊕⊕ High	
**LDL-C/HDL-C - High dose Subgroup**												
4	randomised trials	not serious	not serious	not serious	not serious	none	93	84	-	MD **0.06 higher** (0.64 lower to 0.75 higher)	⊕⊕⊕⊕ High	
**ApoB**												
2	randomised trials	not serious	not serious	not serious	not serious	none	60	50	-	MD **0.03 lower** (0.07 lower to 0.02 higher)	⊕⊕⊕⊕ High	
**ApoA**												
2	randomised trials	not serious	not serious	not serious	not serious	none	60	50	-	MD **0.04 lower** (0.09 lower to 0)	⊕⊕⊕⊕ High	

CI: confidence interval; **MD:** mean difference

Author(s):
Question: Serum glycemia compared to placebo for [health problem]
Setting:
Bibliography: . [Intervention] for [health problem]. Cochrane Database of Systematic Reviews [Year], Issue [Issue].

Certainty assessment							№ of patients		Effect		Certainty	Importance
№ of studies	Study design	Risk of bias	Inconsistency	Indirectness	Imprecision	Other considerations	Serum glycemia	placebo	Relative (95% CI)	Absolute (95% CI)		
**Glucose**												
2	randomised trials	not serious	serious	not serious	not serious	none	61	60	-	MD **0.12 higher** (1.69 lower to 1.92 higher)	⊕⊕⊕◯ Moderate	
**Insulin**												
1	randomised trials	not serious	not serious	not serious	not serious	none	39	38	-	MD **0.63 lower** (0.96 lower to 0.31 lower)	⊕⊕⊕⊕ High	
**HOMA-IR**												
1	randomised trials	not serious	not serious	not serious	not serious	none	39	38	-	MD **0.23 lower** (0.31 lower to 0.16 lower)	⊕⊕⊕⊕ High	

CI: confidence interval; **MD:** mean difference

Author(s):
Question: Phospholipid compared to placebo for [health problem]
Setting:
Bibliography: . [Intervention] for [health problem]. Cochrane Database of Systematic Reviews [Year], Issue [Issue].

Certainty assessment							№ of patients		Effect		Certainty	Importance
№ of studies	Study design	Risk of bias	Inconsistency	Indirectness	Imprecision	Other considerations	Phospholipid	placebo	Relative (95% CI)	Absolute (95% CI)		
**PL**												
3	randomised trials	not serious	not serious	not serious	not serious	none	72	72	-	MD **0.04 lower** (0.1 lower to 0.02 higher)	⊕⊕⊕⊕ High	
**PC**												
3	randomised trials	not serious	not serious	not serious	not serious	none	72	72	-	MD **0.06 lower** (0.1 lower to 0.02 lower)	⊕⊕⊕⊕ High	
**Lyso-PC**												
3	randomised trials	not serious	not serious	not serious	not serious	none	72	72	-	MD **0** (0.01 lower to 0.01 higher)	⊕⊕⊕⊕ High	
**PE**												
3	randomised trials	not serious	serious	not serious	not serious	none	72	72	-	MD **0.02 lower** (0.04 lower to 0)	⊕⊕⊕◯ Moderate	
**PI**												
3	randomised trials	not serious	serious	not serious	not serious	none	72	72	-	MD **0.1 lower** (0.18 lower to 0.01 lower)	⊕⊕⊕◯ Moderate	

CI: confidence interval; **MD:** mean difference

Author(s):
Question: Enzyme compared to placebo for [health problem]
Setting:
Bibliography: . [Intervention] for [health problem]. Cochrane Database of Systematic Reviews [Year], Issue [Issue].

Certainty assessment							№ of patients		Effect		Certainty	Importance
№ of studies	Study design	Risk of bias	Inconsistency	Indirectness	Imprecision	Other considerations	Enzyme	placebo	Relative (95% CI)	Absolute (95% CI)		
**AST**												
2	randomised trials	not serious	not serious	not serious	not serious	none	53	53	-	MD **0.58 higher** (0.54 lower to 1.71 higher)	⊕⊕⊕⊕ High	
**ALT**												
2	randomised trials	not serious	not serious	not serious	not serious	none	53	53	-	MD **1.16 higher** (1.18 lower to 3.51 higher)	⊕⊕⊕⊕ High	
**CRP**												
2	randomised trials	not serious	not serious	not serious	not serious	none	50	51	-	MD **0.21 higher** (1.17 lower to 1.59 higher)	⊕⊕⊕⊕ High	

CI: confidence interval; **MD:** mean difference
